# Immature Reticulocyte Fraction and Absolute Neutrophil Count as Predictor of Hemopoietic Recovery in Patients with Acute Lymphoblastic Leukemia on Remission Induction Chemotherapy

**DOI:** 10.4274/tjh.2014.0379

**Published:** 2016-05-16

**Authors:** Shan E. Rauf, Saleem Ahmed Khan, Nadir Ali, Nabeel Khan Afridi, Maria Haroon, Ammara Arslan

**Affiliations:** 1 Armed Forces Institute of Pathology, Department of Hematology, Rawalpindi, Pakistan

**Keywords:** Acute lymphoblastic leukemia, Lymphoid cell neoplasm, Hematopoiesis, Chemotherapy

## Abstract

**Objective:**

Acute lymphoblastic leukemia (ALL) encompasses a group of lymphoid neoplasms that are more common in children and arise from B-and T-lineage lymphoid precursor cells. The immature reticulocyte fraction (IRF), a new routine parameter in hematology analyzers, can give an indication of hemopoietic recovery like absolute neutrophil count (ANC). The purpose of this study was to evaluate IRF in excess of 5% was considered as IRF recovery.

**Materials and Methods:**

In this descriptive study, 2.5 to 3 mL of EDTA blood of 45 ALL patients undergoing the remission induction phase of their treatment was sampled and analyzed with a Sysmex XE-5000 on day 1 and every second day thereafter until the day of recovery. ANC of >0.5x109/L on the day corresponding to the first of the three consecutive counts was considered as the day of ANC recovery. IRF recovery was an IRF in excess of 5%.

**Results:**

The mean age of the patients was 12.04±5.30 years; 25 patients (55.6%) were male and 20 patients (44.4%) were female. On day 1 of induction remission, the mean IRF value was 9.68±1.41, while the mean ANC value was 0.077±0.061. Mean recovery day for IRF was 11.84±7.44 and mean recovery day for ANC was 17.67±8.77 (two- tailed p-value <0.0001 with 95% confidence interval). By day 28, out of 45 patients 36 (80%) showed ANC recovery, while 41 (91%) showed IRF recovery. The remaining patients who had not shown recovery by day 28 were further followed up and all of them showed recovery of both parameters by day 39.

**Conclusion:**

This study concluded that postinduction bone marrow hemopoietic recovery was earlier by IRF than ANC in children with ALL on chemotherapy.

## Abstract (tr)

**Amaç:**

Akut lenfoblastik lösemi (ALL) çocuklarda daha sık görülen ve B- ve T-lenfoid öncül hücre dizilerinden kaynaklanan bir grup lenfoid neoplaziyi kapsamaktadır. Hematolojik incelemede yeni rutin bir parametre olan immatür retikülosit fraksiyonu (İRF), mutlak nötrofil sayısı (MNS) gibi hematopoietik toparlanma hakkında fikir oluşturabilir. Bu çalışmanın amacı remisyon indüksiyon kemoterapisi almakta olan ALL hastalarında İRF ve MNS toparlanmasını değerlendirmektir.

**Gereç ve Yöntemler:**

Bu tanımlayıcı çalışmada, remisyon indüksiyon tedavisi alan 45 ALL hastasından tedavinin birinci günü başlayarak, toparlanmaya kadar günaşırı 2,5-3 mL EDTA’lı tüpe kan alınmış ve Sysmex XE-5000 ile inceleme yapılmıştır. MNS toparlaması, ardışık üç sayımda MNS değerinin >0,5x109/L olduğu günlerden ilki olarak tanımlanmıştır. İRF toparlanması İRF’nin %5’i aşması olarak belirlenmiştir.

**Bulgular:**

Hastaların ortalama yaşı 12,04±5,30 yıldı; 25 hasta (%55,6) erkek ve 20 hasta (%44,4) kadındı. İndüksiyon tedavisinin birinci gününde, ortalama İRF değeri 9,68±1,41 iken, ortalama MNS değeri 0,077±0,061 idi. İRF için ortalama toparlanma süresi 11,84±7,44 gün ve MNS için ortalama toparlanma süresi ise 17,67±8,77 gündü (p değeri <0,0001, %95 güven aralığı içinde). Yirmi sekizinci günde, 45 hastanın 36’sında (%80) MNS toparlanması varken 41’inde (%91) İRF toparlanması bulunmaktaydı. Yirmi sekizinci günde toparlanması bulunmayan hastaların takibine devam edildi ve 39. günde bu hastaların tamamında her iki parametre açısından da toparlanma tespit edildi.

**Sonuç:**

Bu çalışma kemoterapi alan ALL’li çocuklarda indüksiyon tedavisi sonrasında kemik iliği hematopoietik toparlanmanın İRF’de MNS’ye göre daha erken olduğunu göstermiştir.

## INTRODUCTION

Leukemia is the most common malignancy of childhood and acute lymphoblastic leukemia (ALL) accounts for up to 75% to 80% of the leukemia cases in the world [1]. In the Pakistani population, the frequency of ALL in children and adults combined is 32% of all malignancies [2]. The first case of leukemia in an adult was reported in 1845 by John Hughes Bennett and in children by Henry Fuller in 1846 [2]. Over the last 50 years many new modalities of diagnosis and treatment of leukemia have evolved, leading to improved survival [3,4].

Chemotherapy is the initial treatment of choice in most patients of ALL and is divided into the following stages: remission induction, consolidation or intensification, and maintenance (continuation) therapy, with central nervous system prophylaxis generally provided in each stage. The aim of remission induction therapy is to induce a complete remission. The initial response to remission induction therapy is one of the most important prognostic factors in ALL [5]. The cytotoxic chemotherapeutic agents cause marrow suppression, making patients prone to anemia, bleeding, and infections. The main cause of death in two-thirds of the patients is infection, mostly fungal [6].

During the period of marrow suppression, extensive monitoring of blood counts is required to assess hemopoietic recovery. The hemopoietic recovery can be assessed by conventional parameters like absolute neutrophil count (ANC) recovery or the newer but less commonly used parameter of immature reticulocyte fraction (IRF) recovery. The ANC has >96% sensitivity to predict bone marrow recovery after chemotherapy [7]. The IRF is now being widely used in different centers as an early predictor for hemopoietic recovery in place of the more traditional parameter of ANC recovery, which appears later in the induction phase. According to a prior study, IRF shows early bone marrow recovery in 78% of cases as compared to ANC [8] and has a sensitivity of 92% [9]. Reticulocytes reflect the erythropoietic activity of the bone marrow and were traditionally assessed by manual microscopic method; however, this method is subject to high variability. Today it is measured more objectively by flow cytometry-based hematology analyzers, which measure the messenger ribonucleic acid (RNA) content and the maturity of reticulocytes. The fluorescence of reticulocytes is dependent on the RNA content of the reticulocytes. Immature reticulocytes with higher RNA content will have maximum fluorescence, while reticulocytes with lower RNA content produce minimum fluorescence. On the basis of fluorescence intensity signals, reticulocytes are classified into 3 maturation stages: low-fluorescence reticulocytes (LFRs), medium-fluorescence reticulocytes (MFRs), and high-fluorescence reticulocytes (HFRs). The IRF is the combination of HFRs and MFRs and its fraction in excess of 5% is postulated as a reliable marker for hemopoietic recovery [8].

Flow cytometry-based hematology analyzers are now being used in most of the large diagnostic centers of Pakistan, whereas assessment of hemopoietic recovery is still based on the conventional parameter of ANC. Unfortunately, due to lack of published data in regard to the importance of the IRF during treatment of ALL patients, this parameter is not being used effectively to monitor patients’ marrow status and so far has not been used as a protocol in Pakistan. The aim of this study is to evaluate IRF as an earlier indicator of bone marrow recovery than ANC in patients with ALL on remission induction chemotherapy.

## MATERIALS AND METHODS

This descriptive study was carried out in the Department of Hematology at the Armed Forces Institute of Pathology, Rawalpindi, over a period of 1 year from January 2013 to January 2014. Sampling was done based on a consecutive non probability sampling technique. All diagnosed ALL patients undergoing remission induction chemotherapy of both genders were included. Remission induction as per the UKALL 2003 protocol was given, comprising dexamethasone, vincristine, L-asparaginase, and intrathecal methotrexate. Relapsead patients and those undergoing reinduction were excluded. For each included patient, 2.5 to 3 mL of EDTA anticoagulated blood was sampled and analyzed on day 1 and every second day thereafter until the day of recovery. For each sample of blood, complete blood counts along with differential leukocyte count (for calculation of ANC) and reticulocyte parameters were noted after running the sample on the flow cytometry-based hematology analyzer Sysmex XE-5000. ANC of more than >0.5x109/L on the day corresponding to the first of three consecutive scores was considered as ANC recovery. IRF recovery was an IRF (MFR+HFR) in excess of 5%.

As a control, 2.5 to 3 mL of EDTA anticoagulated blood of normal healthy individuals was also examined for reticulocyte parameters on the Sysmex XE-5000. All the collected data were analyzed with SPSS 19.0. The mean and standard deviation were calculated for quantitative variables like age, ANC, and IRF, and comparisons of means were carried out by paired samples t-test. For qualitative variables like gender, frequency and percentage was calculated.

## RESULTS

A total of 45 patients were included in this study. The majority of the patients were 11-20 years of age. Mean age of the patients was 12.04±5.30 years, and 25 patients (55.6%) were male and 20 patients (44.4%) were female.

**Figure 1:**
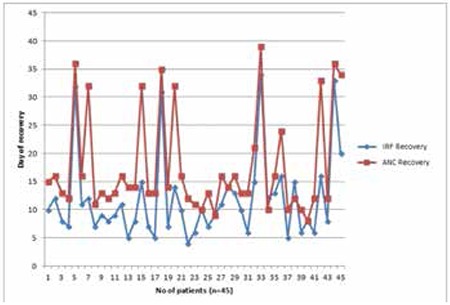
Recovery day of each patient by both immature reticulocyte fraction and absolute neutrophil count.

Mean IRF value on day 1 of induction remission was 9.68±1.41, mean ANC value on day 1 of induction remission was 0.077±0.061, mean recovery day for IRF was 11.84±7.44, and mean recovery day for ANC was 17.67±8.77 (two-tailed p-value <0.0001, 95% confidence interval). Mean values of different variables of ANC and IRF are shown in Table 1. Out of 45 patients, 40 (88.9%) patients showed earlier IRF recovery as compared to ANC. Four (8.9%) patients had the same day of recovery by both IRF and ANC while one (2.2%) had a later IRF recovery than ANC. The recovery days of each patient for both ANC and IRF are shown in Figure 1. By day 28, 41 (91%) patients showed IRF recovery, and ANC recovery was seen in 36 (80%) patients. All those patients not showing recovery by day 28 were further followed up and all of them showed recovery by day 39, as shown in Table 1 and Figure 1.

## DISCUSSION

Chemotherapy for ALL has not changed much over the years, except for a few variations in different centers. The chemotherapeutic agents used in remission induction therapy usually cause severe myelosuppression in these patients. This critical period is variable in patients and requires critical care, supportive therapy, and regular monitoring. Many patients succumb to severe sepsis and bleeding in this period. Recovery of bone marrow from myelosuppression is an indicator of likely hematological remission. The ANC has traditionally been used as an early predictor of bone marrow recovery. However, with the advent of the latest flow cytometry-based hematological analyzers, the IRF is being increasingly used for this purpose.

The IRF is an accurate and reliable parameter easily obtained from automated cell counters such as the Sysmex XE-Series [9]. Several studies showed that the immature reticulocytes detected by flow cytometry are earlier indicators of bone marrow recovery than the detection of ANC in post chemotherapy patients with acute leukemia [10,11].

Although our study was limited in time and sample size, we were able to reach similar conclusions to those published by George et al., who found that immature reticulocytes indicate engraftment, and the use of immature reticulocytes might enable the cessation of antibiotics and growth factors, which could lead to earlier discharge from the hospital and cost savings [9].

The Spanish Multicentric Study Group for hemopoietic recovery also concluded that a rise in IRF indicates hemopoietic recovery [12] and IRF recovery was seen in 91.2% of ALL patients on remission induction before ANC recovery. Luczynski et al. in their study stated that IRF was the first sign of hemopoietic recovery and might be used as a parameter of bone marrow function in clinical studies [13].

Das et al. in 2006 also showed IRF as the earlier predictor of bone marrow recovery as compared to ANC in childhood malignancies [10]. The median day for IRF recovery was 21, while for ANC recovery it was 23 [12]. In a study done in Bangladesh, IRF showed 78% similar or earlier recovery in patients with ALL on remission induction chemotherapy. The mean day for IRF recovery was 16.6±4.6 while it was 23.3±5.7 for ANC recovery, indicating that IRF is an earlier predictor of bone marrow hemopoietic recovery than ANC [8].

In our study, mean recovery days were 11.84±7.44 for IRF and 17.67±8.77 for ANC, showing earlier IRF recovery by an average of 6 days, while it was shown to be 4 days earlier by Grazziutti et al. [14]. This prediction of early recovery by the simple and reproducible parameter of IRF can have significant impact on the management of patients.

## CONCLUSION

This study concluded that the IRF shows earlier hemopoietic recovery as compared to the current practice of ANC for the monitoring of ALL patients on remission induction chemotherapy.

This early laboratory indicator of hemopoietic recovery will guide clinicians to make early and important therapeutic decisions in such patients. Nowadays, the IRF is offered by most new hematology analyzers. Moreover, this test is a simple, quick, reproducible, and reliable parameter in the automated hematology analyzers.

## Figures and Tables

**Table 1:**
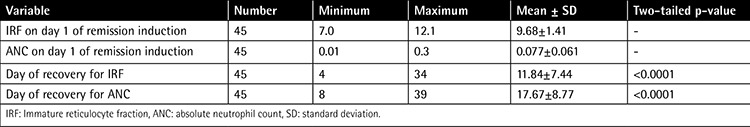
Mean values of different variables.
